# Furious Rabies after an Atypical Exposure

**DOI:** 10.1371/journal.pmed.1000044

**Published:** 2009-03-17

**Authors:** Heiman F. L Wertheim, Thai Q Nguyen, Kieu Anh T Nguyen, Menno D de Jong, Walter R. J Taylor, Tan V Le, Ha H Nguyen, Hanh T. H Nguyen, Jeremy Farrar, Peter Horby, Hien D Nguyen

## Abstract

Heiman Wertheim and colleagues describe the diagnosis and management of two patients who developed rabies after butchering and consuming a dog or a cat.

## Case Descriptions

**Patient 1.** A 48-year-old male construction worker, with no preceding medical illnesses, was admitted to the intensive care unit of a hospital in Hanoi. For a few days prior to admission he experienced pain and numbness in both forearms and a flushed sensation throughout his body. He also reported increased perspiration and increased libido. At presentation, the patient was lucid but markedly agitated, and was unable to swallow due to involuntary inspiratory muscle spasms when he was presented with a glass of water (Video S1) or when he felt a breeze. On examination he was afebrile, and had a dry mouth, normal heart rate and blood pressure, and a Glasgow Coma Score of 15. He had no focal neurological signs and no neck stiffness, and Kernig's sign was negative. Neither the patient nor his wife recalled that he had been bitten by a dog, cat, bat, or other mammal in the preceding months, and his skin showed no evidence of recent bite injuries or cuts.

**Patient 2.** A 37-year-old male farmer, without any prior medical history, presented himself to a Hanoi outpatient clinic with increased perspiration, intermittent muscle spasms of both legs, and the inability to drink normally due to involuntary inspiratory muscle spasms when he was presented with a glass of water or felt a breeze. Disease onset was several days before admission. On examination, the patient had a normal body temperature, heart rate, and blood pressure, and a Glasgow Coma Score of 15. The patient was lucid with periods of agitation. There were no focal neurological signs and no neck stiffness, and Kernig's sign was negative. He had bilateral pupil dilatation (~5 mm) with weak direct and consensual light reflexes. There were no signs of recent bites or other skin injuries, but the patient reported that he had been bitten in the heel by a pet dog one month prior to presentation. The dog had remained healthy since the bite.

The families of the patients in this manuscript have given written informed consent to publication of their case details.

## At This Stage, What Was Our Differential Diagnosis?

Both patients presented with an acute progressive encephalopathy in which a prominent symptom was involuntary inspiratory muscle spasms when exposed to a glass of water (hydrophobia) or draft of air (aerophobia). At presentation there was no fever, and from the medical history it was unclear whether the patients had fever prior to presentation. There were no clinical signs of meningism that supported a diagnosis of bacterial meningitis. Tetanus was considered unlikely as the characteristic muscle rigidity and trismus were absent in both patients [1]. Neither patient had been vaccinated recently, ruling out post-vaccinal encephalomyelitis (acute disseminated encephalomyelitis), which may occur following vaccination [2]. Encephalopathy due to intoxication is usually associated with cognitive dysfunction and hence was considered unlikely as both patients had good cognitive function at presentation.

Acute progressive encephalitis can be caused by several neurotropic viruses, including herpes viruses, enteroviruses, flaviviruses, arboviruses, and rabies virus. Viral encephalitis, other than rabies, can cause behavioural changes, but not the hydrophobic spasms that were observed in both patients [3]. Hydrophobia is specific for encephalitic rabies and is considered pathognomonic for this disease [4].

Rabies can manifest itself in three forms: (1) classic encephalitic (furious) rabies, (2) paralytic (dumb) rabies, and (3) non-classic atypical rabies. All forms are progressive and generally lead to death [5]. The majority of the cases present as encephalitic rabies, with hydrophobia and hyper-excitability. Paralytic rabies presents with flaccid muscle weakness, and can be confused with Guillain-Barré syndrome, although urinary incontinence and ongoing fever can distinguish paralytic rabies from Guillain-Barré syndrome [3]. Paralytic rabies can also present as a febrile encephalopathy, requiring laboratory testing to help identify the cause. The pathogenesis underlying these two clinical forms remains to be elucidated. The non-classic atypical rabies usually occurs following exposure to the bite of a bat, but has also been described after dog bites and presents amongst other neurological symptoms with neuropathic pain, focal brainstem signs, and myoclonus [5].

## Why Did We Think That Rabies Was the Most Likely Diagnosis?

Both patients presented with hydrophobia, typical for furious rabies, which is induced by attempts to drink water, leading to reflex contraction of the inspiratory muscles and an inability to swallow [6]. Similar reflexes can be elicited by air flow (aerophobia) and other stimuli like noise [6]. These symptoms are caused by destruction of brain stem neurons that inhibit the inspiratory motor neurons [6,7]. Patients can have an inexplicable terror of water, associated with hydrophobia [7]. Due to decreased fluid intake, most patients have moderate to severe dehydration and renal impairment [8].

As hydrophobia is pathognomonic for rabies, other causes of encephalitis were considered unlikely. A typical exposure, like a dog bite, is not required in order to establish a diagnosis of rabies, as patients regularly do not recall such exposures, which may have occurred weeks to months before, and some exposures may go unnoticed. Neither patient had a history of an animal bite that may have been the source of infection. For this reason, neither patient received post-exposure rabies prophylaxis. Patient 1 did not have an exposure history that would require rabies prophylaxis as recommended by the World Health Organization (Table 1), and patient 2 was bitten by a dog that had remained well in the intervening months. World Health Organization guidelines state that if a dog has remained well for ten days after the exposure, there is little risk for rabies transmission and post-exposure treatment can be stopped [4]. However, both patients did have an alternative exposure that could have been a source of rabies infection.

**Table 1:**
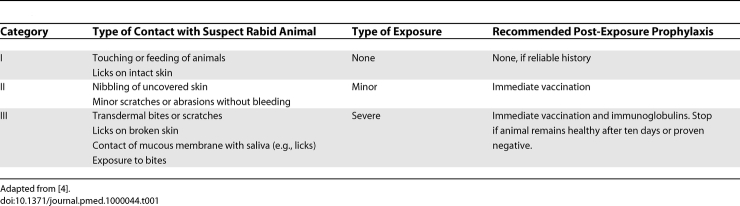
Recommended Post-Exposure Prophylaxis, Depending on Type of Exposure

**Patient 1.** Two months before admission, the patient had butchered and consumed a dog that had been killed in a road traffic accident. The patient took the dog's carcass home where he first extracted all the teeth with a knife. He mentioned he did this as a preventive measure against rabies, as he was aware of the presence of rabid dogs in his neighbourhood. He then singed the hide to remove the hair. This was followed by opening the skull to remove the brain, which was then steamed in leaves and eaten. During this butchering, the patient wore workman's gloves but no other protective equipment. The patient did not recall receiving any cuts or other injuries during preparation of the dog. Others who ate parts of the same dog remained well. All parts of the dog were cooked prior to being eaten.

**Patient 2.** Three weeks before admission the patient had killed, butchered, and consumed a cat that had been sick for three days. The cat had showed altered behaviour by sitting quietly in a dark corner, meowing and not wanting to be disturbed. The patient killed the cat by battering it, after which he singed the hide to remove the hairs. This was followed by opening the skull to remove the brain which the patient pulped with his bare hands to make a Vietnamese dish, called “rua man”. This dish, consisting of cooked cat meat and other organs, was also consumed by other individuals who all remained well.

The exposure of both patients to the dog and cat, respectively, occurred within the recognised incubation period for rabies, which is most commonly one to three months after the exposure [3]. In Viet Nam, dog butchering is considered by clinicians to be a risk factor for rabies transmission.

## Which Diagnostic Tests Are Now Helpful?

A lumbar puncture to obtain cerebrospinal fluid (CSF) was performed on both patients. The CSF showed mild pleocytosis and a normal glucose and protein level (Table 2), which is consistent with a diagnosis of rabies, although most rabies patients do have a mild elevated protein level in their CSF [3].

**Table 2:**
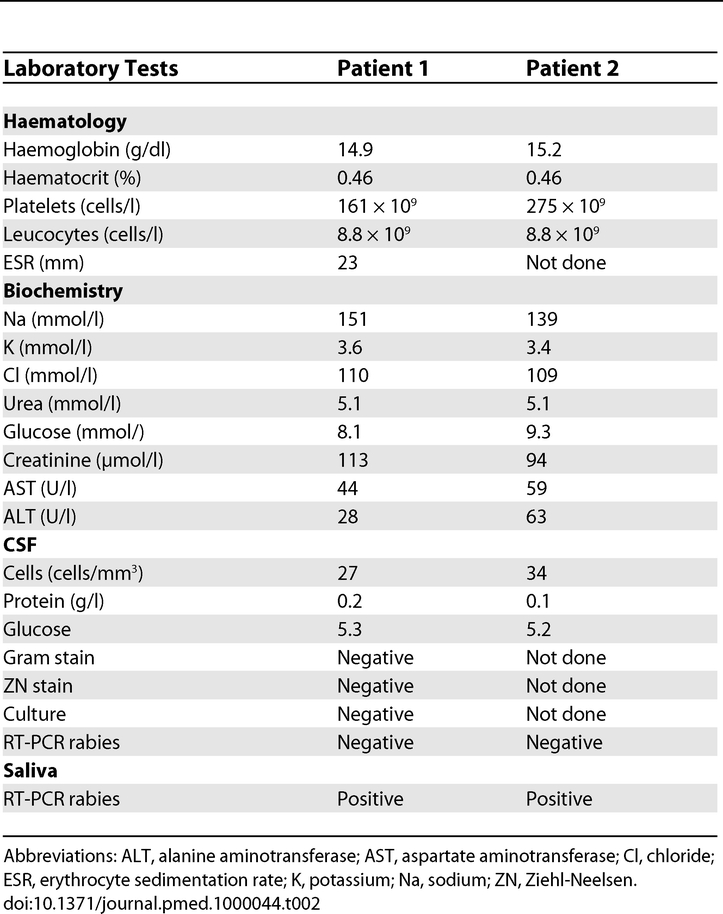
Laboratory Results on Admission, Including Microbiology

Several tests are available to confirm the diagnosis of rabies: reverse transcriptase polymerase chain reaction (RT-PCR), serology, direct fluorescent antibody test, histology, and viral culture [4,9]. Suitable specimens for rabies detection are saliva or a buccal swab, skin biopsy from the nape of the neck (containing hair follicles with nerve endings), ante- or post-mortem brain biopsy, CSF, and urine. In Asia and Africa, brain biopsies are rarely approved by the patient's family [10]. Serology can be performed on serum and CSF, but is of little immediate diagnostic value in the acute stage of the disease as rabies antibodies are generally not formed before the second week of illness [9,11,12]. Serology can be helpful for late diagnosis, for the purpose of instituting appropriate infection control and public health measures.

Molecular techniques, like RT-PCR, for rabies are becoming more widely available in developing countries [10]. Providing laboratories should have stringent procedures to prevent contamination. Suitable diagnostic specimens for detecting rabies virus RNA are saliva, buccal swab, skin (nape of the neck) and brain biopsy, and, to a lesser extent, CSF and urine [10,13,14]. Testing sequential specimens is sometimes necessary to ensure rabies diagnosis, but obtaining specimens can be difficult as patients deteriorate rapidly.

Both patients had positive RT-PCR results from their saliva but not from their CSF samples, using a previously described technique [15]. Serology was negative for both patients using a commercial ELISA (Platelia Rabies II kit, Biorad; http://www.bio-rad.com/).

## What Is the Meaning of a Positive Rabies RT-PCR?

The RT-PCR positive test results from the saliva specimens of both patients confirmed the clinical diagnosis of rabies. Other than brain and skin biopsies, saliva has the highest rate of positivity, of approximately 75% [10,16]. Saliva can reach a 100% sensitivity when three successive samples are tested [10,16]. A recent study showed that RT-PCR on skin biopsies reached more than 98% sensitivity and 100% specificity, which correlates well with the gold standard of the immunofluorescence test on brain biopsies [10]. The diagnostic value of urine is limited, as less than 10% of rabies patients have a RT-PCR positive urine at admission [10]. The sensitivity of buccal swabs is lower than that of saliva [10]. A false-positive result may occur because of poor laboratory technique and contamination, and hence stringent operating procedures must be in place and appropriate controls should be included at every stage from extraction of the nucleic acid through to the amplification stages.

Neither patient had rabies antibodies in sera obtained within a week of disease onset, as this was too soon for seroconversion [11,12]. Specimens could not be obtained at a later stage as the patients died soon after admission.

## What Is the Standard Management for This Condition?

Rabies is almost invariably fatal [17]. A small number of survivors have been reported, but all except one had received some form of pre- or post-exposure prophylaxis [17,18]; this patient, who developed rabies after being bitten by a bat, was treated with a combination of supportive care, a drug-induced coma, and antiviral treatment with ribavirin and amantadine, known as the Milwaukee protocol [18]. Attempts with this experimental regimen in other patients have been unsuccessful, and all died [19]. Currently the efficacy of this experimental treatment remains unclear and requires further study.

Rabies comes with agonizing symptoms, and therefore good supportive and palliative care remains the cornerstone of rabies management [17,20]. Though a well-documented case of human-to-human transmission of rabies has not been described and is considered rare, it is advised to use barrier precautions for those taking care of the patient. Neurological symptoms and medical complications of the rabies patient need to be anticipated and can partly be managed with the use of sedatives, narcotic analgesics, antiepileptic drugs, and neuromuscular blockers [17]. Haloperidol with or without diazepam was found to improve palliative care in the Philippines, but the efficacy differs per patient and requires tailoring [20]. Unfortunately, practical guidelines for resource-poor settings are unavailable. There is a need for recommendations on how to provide palliative care for rabies patients in these settings. Because patients are often taken home to die by their relatives, to avoid perceived unnecessary costs, recommendations for palliative care also need to be applicable to the home setting [10].

## What Was the Outcome of the Described Cases?

Patient 1 received supportive treatment with diazepam and intravenous rehydration, as is standard practice in Viet Nam. Several hours after admission, the patient developed a fever of 39 °C and became progressively more agitated and restless. For this reason, coma was induced by thiopental and mechanical ventilation started. His fever persisted, and he became hypotensive and developed acute renal failure. Six days after admission, the patient was taken home by his family to die. Patient 2 was taken home by his family on the day of admission after the clinical diagnosis was made, because of the poor prognosis. He died one day after discharge.

## Discussion

Here we present two patients with laboratory-confirmed rabies who became symptomatic after butchering, preparing, and consuming a dog and a cat. The point of entry of the rabies virus in these cases is unclear, but removal and preparation of the dog's and cat's brains may have generated large amounts of infectious rabies virus, with transmission occurring via either the conjunctiva, or the oral and nasopharyngeal mucosae [21]. Alternatively, the patients may have become infected through contamination of unrecognized cuts or abrasions of their hands. Rabies following the handling of infected carcasses has been previously reported [22]. Another possibility is oral transmission, as rabies has also been shown to be transmitted orally in experiments [23–25]. But consumption of infected brain seems a less likely route of infection because, in both cases, the brain meals were well cooked and shared with other people who did not develop rabies.

Since we had no access to specimens from the butchered and consumed animals and were unable to test them for rabies, the exact source of rabies in these patients remains uncertain, and another source of unnoticed animal contact cannot be excluded. However, both patients became ill within the expected incubation time for rabies following the exposure during butchering, and the butchered cat showed abnormal behaviour before being killed, consistent with the prodromal stage of rabies. No information was available on the health of the dog prior to its death, but rabies could have made it accident-prone, resulting in the accident which killed it.

**Figure 1:**
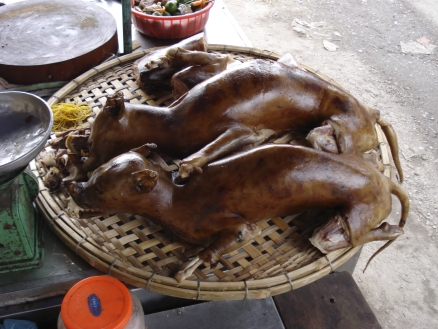
Dog Meat for Consumption for Sale at a Market in Hanoi, Viet Nam

Rabies is estimated to cause 31,000 deaths per year in Asia, representing approximately 60% of the annual cases worldwide [26], and the number of human cases has been increasing in China and Viet Nam [27]. This number is an underestimate, as only encephalitic rabies is recognized, while other forms are often missed [28]. In China, where fewer than 10% of dogs are vaccinated, 3,380 people are reported to have died of rabies in 2007 [29]. Eating dog meat (Figure 1) and, to a lesser extent, cat meat is common in many Asian countries, including Viet Nam, South Korea, the Philippines, Laos, Myanmar, Cambodia, Thailand, India, Kazakhstan, and the People's Republic of China. In Viet Nam, the consumption of dog meat is more common in the northern provinces. It is believed that the eating of dog meat “enhances health and longevity” [30]. Dog meat is consumed throughout the year in the second half of the lunar month, and is more popular during the winter months, as it is also believed to increase body heat [30].

In 2007, ten human rabies cases (80% male and all older than 15 years) were confirmed by the laboratory of the National Institute of Hygiene and Epidemiology of Viet Nam (NIHE). Of these ten cases, four (40%) did not have a history of dog bites, and three of these four had prepared dog meat from sick animals prior to onset of illness. The fourth patient did not report being bitten by a dog or handling dog meat, but had eaten dog meat. NIHE conducted a study in dog slaughter houses in the Hanoi area in 2007 and found that two out of ten (20%) sick dogs were positive for rabies [31]. Vietnamese doctors consider dog butchering a risk factor for rabies transmission.

Slaughtering of dogs has been reported as a risk for rabies transmission in the Philippines and in China [32,33]. In January 2008, 30 people from the Philippines were reported to have received anti-rabies vaccination after having eaten the meat of a rabid dog [32]. An epidemiological survey in China reported that two out of 64 patients contracted rabies by either killing, cooking, or consuming dogs [33,34]. These data illustrate that the risk of transmission through butchering and processing of rabid animals is not restricted to Viet Nam. Slaughtering of unvaccinated rabies reservoir species in endemic areas needs to be considered a category III exposure (Table 2), requiring prophylaxis.

**Key Learning Points**

- Butchering of unvaccinated dogs and cats in rabies-endemic countries should be considered a risk factor for rabies transmission.
- The diagnosis of classic furious rabies presenting with hydrophobia can be made clinically by experienced doctors. Paralytic rabies, atypical rabies, and the prodromal stages of classic furious rabies are less specific, and laboratory testing is required.
- Antigen detection by molecular (RT-PCR) and immunofluorescent techniques are used to confirm diagnosis, preferably on two or more specimens, like skin biopsy from the nape of the neck, saliva, or buccal swab.
- Rabies is a fatal disease. The few described survivors generally have serious neurological sequelae. A recent survivor did have a favourable outcome after an experimental therapy, which requires further study.
- Good supportive and palliative care is the cornerstone of rabies case management. Rabies case management guidelines for resource-poor settings need to be developed and implemented.

Neither of the patients we describe sought or received potentially life-saving post-exposure rabies prophylaxis. Rabies control programmes should carry out public health messaging campaigns to alert clinicians and the general public that the butchering and handling of meat from unvaccinated reservoir species is a risk factor for acquiring rabies. Dogs are the main reservoir for human rabies, and the raising, butchering, processing, and consumption of dogs should be regulated and controlled. In Viet Nam, the national program for rabies control and prevention includes workers at dog slaughterhouses in their vaccination program. However, the private slaughter of dogs is common in Viet Nam, and individuals involved in this practice will not be captured in this program. Unregulated raising and slaughtering of dogs should be rigorously discouraged through legislation and education. Ultimately, the best way to prevent rabies is to vaccinate the reservoir species.

## Supporting Information

Video S1 Patient 1 with Hydrophobia Oral and written informed consent from the patient and his family was obtained. (4221 KB WMV).

## Abbreviations

CSF: cerebrospinal fluid
RT-PCR: reverse transcriptase polymerase chain reaction
